# Systemic Management of Peridontoclasia

**Published:** 1920-08

**Authors:** Elmer S. Best


### SYSTEMIC MANAGEMENT OF PERIDONTOCLASIA

BY ELMER S. BEST, D.D.S.

It is my belief that periodontia includes but a part of the service which is usually found necessary in the majority of cases. While it occupies first place in treatment of infections of the mouth, it is but a part of the work necessary in restoring health and function. In order that we may comprehend fully our responsibility in treatment of complex cases, it has been found advisable to divide our work into three separate and distinct procedures, all of which are equally important to the patient and are directly influenced by a correct diagnosis, which should be made for each case. It is our practice to commence each case by making a standardized examination which consists of the following:

### STANDARDIZED EXAMINATION

1. Complete radiographic examination.
2. Vitality test, both trans-illumination and electric.
3. Study casts. These are standard orthodontic casts.
4. Clinical examination of the teeth and periodontium.

All of this data is carefully recorded upon charts and filed. Included with this, we also have the case history. All this is prior to the first operation. As the treatment of the case progresses, a record is made, from time to time, of each change of condition as it occurs. Thus, it will be seen that the indications for treatment of cases are founded upon the clinical evidence revealed in the examination. It would seem that a logical procedure would be:

1. To bring about as rapidly as possible a sanative influence upon the entire mouth. This may be accomplished in part by the usual prophylactic procedure.

2. The extraction of hopelessly diseased teeth. It will be admitted that complications incident to exodontia are greatly minimized in a mouth which has been prepared.

3. The correction of traumatic occlusion. It would seem unnecessary to describe our technique in this.

4. The introduction of correct use of the tooth brush. In the resolving of disease and the restoration of tissue tone, the tooth brush is a most important factor.

5. Treatment of infected dentin. By this is meant treatment of caries, either cervical or coronal, ccurring in vital teeth, special attention being given to those cases where caries has endangered the vitality of the pulp. Brevity necessitates that a detailed description of this procedure be omitted in this paper. The writer, however, attaches great importance to it as a factor in the successful management of cases.

6. The necessary treatment and filling of root canals. If pulpless teeth are to be retained, it should be remembered that it is only possible in the absence of infection within the canals and periapical regions. This is a phase of periodontia which the writer believes to be very frequently neglected, to the detriment of the patients and to the discredit of the periodontist.

To epitomize the above, it will be seen, that we have located, recorded and removed the major portion of those factors which are responsible for the degenerative processes in the development of the pathologic changes in the periodontium. Attention is called to the fact that this has necessitated several visits and an elapsed time of possibly two or three weeks. Also, that up to this time no extensive surgical procedure upon the root surfaces had been instituted. The changes which have taken place in the vascular investing tissues during this time have prepared the case for a sharp regenerative response to the surgical interference. The recognition of this fact has resulted in the establishment of this method of handling cases as routine procedure.

This concludes our consideration of the purely periodontic phase of the type of cases under consideration. It should never be overlooked, however, that this procedure is responsible only for the suppression of the pathological. The necessities of patients demand as a rule extensive restoration of the lost function. The reconstruction problems, whatever their importance, would not properly be discussed in this symposium. They are, however, the second phase of our problem.

Third, lastly, and eternally, it is our duty to maintain that condition of health which has been established. This can only be accomplished by systematic prophylactic measures.—*Dental Item of Interest.*

